# A Self-Administered, Digitized Approach to Quantifying the Cardinal Motor Symptoms in Parkinson’s Disease

**DOI:** 10.3390/s26144497

**Published:** 2026-07-15

**Authors:** Mandy Miller Koop, Colin Waltz, Andrew Bazyk, Brittany Lapin, Yadi Li, Stuart Houltham, Dave Blum, James Liao, Oliver Phillips, Junaid Siddiqui, Andre G. Machado, Jay L. Alberts

**Affiliations:** 1 Department of Biomedical Engineering, Cleveland Clinic, 9500 Euclid Ave., Cleveland, OH 44195, USA; 2 Neurological Institute, Cleveland Clinic, 9500 Euclid Ave., Cleveland, OH 44195, USA; 3 Department of Quantitative Health Sciences, Cleveland Clinic, 9500 Euclid Ave., Cleveland, OH 44195, USA; 4 Ceraxis Health, Inc., 10000 Cedar Avenue, Cleveland, OH 44106, USA; 5 Department of Neurology, Dartmouth Health, 1 Medical Center Drive, Lebanon, NH 03756, USA; 6 Department of Neurosciences, Cleveland Clinic, 9500 Euclid Ave., Cleveland, OH 44195, USA

**Keywords:** Parkinson’s disease, motor assessment, digital assessment, validation, MDS-UPDRS, deep brain stimulation

## Abstract

**Highlights:**

**What are the main findings?**

- Metrics from a self-administered, stylus-based assessment predicted and were significantly correlated with MDS-UPDRS clinical ratings of cardinal PD motor symptoms.
- Metrics from the stylus-based assessment successfully discriminated between PD ON- and OFF-deep brain stimulation (DBS) conditions.

**What are the implications of the main findings?**

- The self-administered, sensor-based platform presented here can be used in-clinic or remotely for accurate, robust evaluation of PD motor symptoms.
- The platform’s demonstrated sensitivity to PD therapy conditions may enhance clinical decision-making in adjusting PD medication and DBS therapies.

**Abstract:**

Many people with Parkinson’s disease (PwPD) lack optimal care due to limited access to neurologists and a reliance on subjective rating scales for treatment decisions. The Ceraxis Insight platform was designed to provide quantitative measures of the cardinal motor symptoms of Parkinson’s disease (PD) through self-administered assessments performed using a tablet paired with a sensor-embedded stylus. The aim of this study was to assess the validity of the Ceraxis Insight outcome metrics against clinical gold-standard measures of PD motor symptoms. Nineteen PwPD completed a clinical examination and the nine Ceraxis Insight assessment modules. Quantitative performance metrics were calculated from the platform’s IMU, force transducer, and touchscreen inputs. Mixed-effect models and correlation analyses determined that multiple quantitative metrics from the Ceraxis Insight modules significantly predicted (*p* < 0.05) and were significantly correlated (correlation coefficients > 0.70) with the MDS-UPDRS III total score, bradykinesia, tremor, rigidity, and postural instability and gait difficulty sub-scores. Logistic regression models determined that multiple Ceraxis Insight metrics discriminated between ON- and OFF-deep brain stimulation (DBS) conditions, with Area Under the Receiver Operating Characteristic Curve (AUC) values exceeding 0.70. The Ceraxis Insight platform provides a validated, objective assessment of PD motor symptoms that may be performed within clinical or remote settings for data-driven evaluation and treatment.

## 1. Introduction

Parkinson’s disease (PD) is a neurodegenerative disease with a rapidly increasing global presence and is expected to affect 12+ million people worldwide by 2040 [1]. The medical management of PD remains challenging due to the complexity and heterogeneity of disease progression and symptom presentation. The cardinal motor symptoms of PD include bradykinesia, tremor, rigidity, as well as postural instability and gait difficulty (PIGD). Bradykinesia (i.e., slowness of movement) is a core symptom, required for a PD diagnosis, and affects nearly all motor activities and everyday tasks [2,3]. Tremor (i.e., rhythmic involuntary movement) affects more than 75% of people with PD (PwPD), and is an episodic symptom that fluctuates in severity throughout the day and the disease process [4,5,6], and is one of the most disruptive PD symptoms as it impacts many daily activities such as eating, dressing, and self-care [4]. Rigidity affects up to 90% of PwPD [7], is among the most painful symptoms of PD, and limits mobility, which can precipitate comorbidities related to sedentary behaviors [8]. Postural instability and gait difficulty are particularly dangerous symptoms that can cause falls and related injuries and are largely refractory to medication or surgical intervention [9]. Given the complexity, multifaceted nature, and substantial impact of PD motor symptoms, effective medical management requires comprehensive evaluation by highly trained clinicians and, often, multiprofessional care.

The American Academy of Neurology and the Parkinson’s Foundation recommend regular visits with a movement disorders neurologist (MDN) for clinical management of PD [10,11] as this has shown to reduce the risk of PwPD transitioning to skilled nursing facilities and lower risks of fractures and even mortality [12]. Traditionally, during these visits, MDNs complete the Movement Disorder Society-Unified Parkinson’s Disease Rating Scale: Part III (MDS-UPDRS III), considered the clinical gold-standard assessment of PD symptom severity [13]. The assessments are prone to subjectivity and are scored on an ordinal (0–4) scale, which has demonstrated limitations in interrater reliability and sensitivity to symptom changes [14,15]. Additionally, due to the physical manipulation required for various assessments, the MDS-UPDRS III exam must be performed in-person. Despite being the standard of care, recent data indicate that less than 10% of PwPD have an annual visit with an MDN, and nearly half do not see any neurologist [16]. The limitations of the MDS-UPDRS III, coupled with the relatively few PwPD under the direct care of an MDN, reveal a fundamental gap in the evaluation and clinical management of PD symptoms. Therefore, improving access and quality of care for PwPD requires a standardized, objective, scalable, and low-cost assessment of motor function.

Over the last decade, numerous technologies have been developed to address the gap in a standardized, objective, and quantitative measure of PD motor symptoms [17]. Several studies have utilized accelerometers or other sensors to quantify a single, isolated symptom of PD [18,19,20,21]. Despite these efforts, a recent systematic review of almost 600 technologies developed to assess PD symptoms found only 6% of systems “reached a technology level that justified the hope of being included in clinical assessments in a useful time period” [22]. The lack of single-platform technology to objectively and comprehensively quantify PD motor symptoms reflects the unrealized potential of leveraging technology to precisely characterize PD motor symptoms.

Using a ‘develop with clinical intent’ approach, the Ceraxis Insight platform was designed to address a critical unmet need in PD by providing a self-administered, standardized tool for evaluating motor symptoms that delivers detailed, objective data to better inform treatment and expand access to high-quality clinical care [23]. The platform contains nine assessment modules that integrate previously validated technologies for quantifying PD postural stability and upper extremity function, and digitize various items of the MDS-UPDRS III, which captures various features of PD cardinal motor symptoms [13,23,24,25]. However, before clinical adoption, the outcome metrics from the Ceraxis Insight platform must be validated against the clinical gold-standard measures. Accordingly, the primary aim of this project was to evaluate the criterion validity of the Ceraxis Insight outcomes against MDN-rated MDS-UPDRS III scores. In addition, this study aimed to evaluate the known-groups validity of the Ceraxis Insight assessments in discriminating between deep brain stimulation (DBS) therapy states.

## 2. Materials and Methods

### 2.1. Participants

Nineteen PwPD participated in the Correlation of Motor Metrics and Neurological Data (COMMAND) study (Table 1). All participants were receiving anti-Parkinsonian medication, and a subset (*n* = 8) had bilateral subthalamic nucleus DBS as part of their PD therapy. Prior to consent, medication status and DBS parameters were clinically optimized and stable for at least six months. Assessments were completed during a single visit to the Cleveland Clinic. Inclusion criteria included (1) a diagnosis of idiopathic PD by an MDN, (2) ability to ambulate 200 m independently, (3) ability to follow 2-step commands, and (4) clinically optimized DBS settings when applicable. Exclusion criteria included (1) a diagnosis of dementia or other (non-PD) neurological condition, or (2) a musculoskeletal injury that significantly altered gait. This study was approved by the Cleveland Clinic Institutional Review Board, and all participants provided informed consent.

### 2.2. Ceraxis Insight Hardware

**Figure 1:**
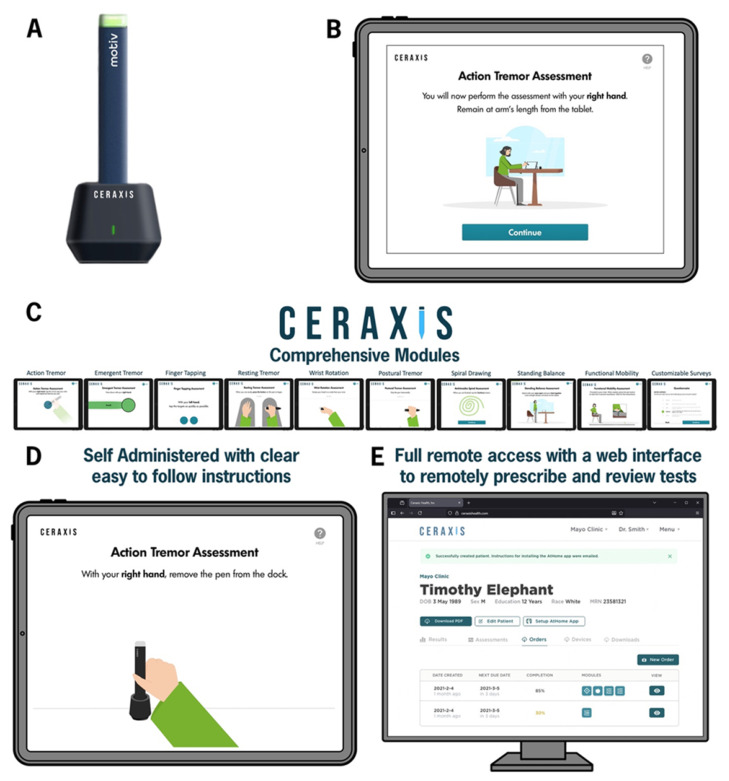
Ceraxis Insight platform and web portal. (**A**) Ceraxis Insight Motiv Stylus with an embedded IMU and force transducer for recording biomechanical data during the assessment modules. (**B**) The Ceraxis Insight application operates on the tablet and includes nine assessment modules. (**C**) The Ceraxis Insight application dashboard provides icons of each of the nine assessment modules and allows participants to navigate through the prescribed assessments. (**D**) Example of the detailed instructions provided to participants at each step of the assessment modules via demonstrations (screenshot shown) and auditory directions. (**E**) Illustration of the Provider Portal interface, which allows providers to view patient information, review scores from completed modules, and prescribe new assessments.

The Ceraxis Insight platform consists of a tablet (iPad; Apple Inc., Cupertino, CA, USA) that runs the user-facing application (Ceraxis Insight application) and a paired stylus (Ceraxis Insight Motiv; Ceraxis Health Inc., Cleveland, OH, USA) (Figure 1). The stylus includes an embedded inertial measurement unit (IMU) consisting of an accelerometer that provides 3D linear acceleration measurements with a resolution and range of 13 bits and +/−2 g, respectively, and a gyroscope that provides 3D angular velocity measurements with a resolution of 16 bits, range of +/− 250 deg/s, and a sensitivity of 14.375 Least Significant Bit/deg/s. The stylus also contains an embedded force sensor in the tip with a maximum displacement of 10 mm (2 kg of force), a Hall effect sensor to detect contact with the stylus base, a button (clicked for trial initiation), and an LED light to indicate active data collection.

### 2.3. Experimental Protocol

All participants completed the Ceraxis Insight assessment modules, the MDS-UPDRS III exam, and the Systems Usability Scale [27]. All testing was performed in the off-medication state (i.e., antiparkinsonian medication withheld 12+ h prior to testing). Participants without DBS (NO DBS) completed the Ceraxis Insight modules, followed by the MDS-UPDRS III. Participants with DBS completed testing both in the ON-DBS and OFF-DBS conditions during one study visit, and this order was randomized. To allow for stabilization of DBS effects, testing began 30 min after the participant’s DBS was turned ON or OFF [28]. The MDS-UPDRS III exam was performed by a board-certified MDN who was blinded from the participant’s therapy states and Ceraxis Insight outcomes. All participants completed the System Usability Scale after all testing was completed.

### 2.4. Ceraxis Insight Assessment Modules

The Ceraxis Insight platform includes nine modules designed to elicit and quantify the cardinal motor symptoms of PD. Seven of the nine modules are utilized to evaluate upper extremity motor symptoms, and two modules evaluate global measures of PIGD, including functional mobility (e.g., postural transitions, gait, and turning) and balance. The Ceraxis Insight modules were adapted from established clinical assessments to accommodate the hardware and workflow features of the platform (Table 2).

### 2.5. Testing Procedure and Data Acquisition

For the upper extremity modules (modules 1–7), participants were seated at a table with the tablet. Before each module, instructions were provided via the tablet, and then participants completed a 5 s familiarization trial of each task to ensure comprehension of the task instructions and requirements. All modules were initiated by the participant: for those involving the tablet (modules 1, 5, 6, and 7), the participant pressed a “begin button” icon on the tablet, and for the stylus-only modules (modules 2, 3, 4, 8, and 9), the participant clicked the button on the stylus to start the trial. The upper extremity trials were completed twice, first with the right hand, then the left, each for 20 s. The Standing Balance module was completed twice, first with eyes open, then with eyes closed, each for 20 s. The Functional Mobility module did not have a set duration: the participant began and ended the assessment by clicking the stylus button immediately before they arose from the chair and after they returned to the seated position.

Data was continuously acquired throughout each module and stored on the tablet. Stylus IMU data were uploaded to the tablet via Bluetooth. A small memory chip was embedded in the stylus to prevent data loss if the Bluetooth connection was lost. Following completion of the testing session, data were uploaded from the tablet to a cloud server and downloaded for offline analysis in MATLAB (version R2024b, The MathWorks Inc., Natick, MA, USA).

### 2.6. Data Processing and Quantification of PD Motor Symptoms

#### 2.6.1. Stylus Recordings

Recordings from the IMU’s accelerometer and gyroscope captured kinematic data with a mean (+/−standard deviation (SD)) sampling rate of 75.4 (+/−28.6) Hz. The 16-bit integers were converted to real-world units. Then the 16-bit Unix time values were converted to a monotonic timestamp using a cumulative sum operation. A force transducer embedded in the stylus tip provided kinetic data. The force transducer recorded time and force with a sampling rate of 22.3 (+/−8.0) Hz.

#### 2.6.2. Tablet Recordings

Time, touch position (x, y), target position (x, y), and target accuracy were recorded at 60 Hz.

#### 2.6.3. Data Processing

All sensor data was downloaded from the cloud to a PC for offline processing using custom MATLAB scripts. Data from the IMU and force transducer were resampled to a uniform sampling rate of 60 Hz and 30 Hz, respectively, using spline interpolation implemented in MATLAB. These data were used to calculate the metrics described below.

### 2.7. Ceraxis Insight Metric Calculations

#### 2.7.1. Bradykinesia Metrics

Quantification of bradykinesia symptoms, including movement speed, amplitude, and rhythmicity, was calculated with kinematic data recorded by the stylus during the Functional Mobility, Kinetic Tremor, and Wrist Rotation modules. In the Kinetic Tremor and Wrist Rotation modules, kinematic data were filtered to isolate voluntary movements from Parkinsonian tremor, canonically in the 4–12 Hz frequency range [29]. Specifically, angular velocity and linear acceleration data from these two modules were filtered using a 4th-order band-pass Butterworth, with cut-off frequencies of 0.25–3.5 Hz [30].

In the Kinetic Tremor module, the amplitude of linear acceleration during voluntary upper extremity movements was quantified utilizing the root mean square (RMS) of linear acceleration [Kinetic Tremor—Voluntary Movement Linear Acceleration RMS (m/s^2^)]. In addition, the maximum value of the angular velocity signal per trial (Kinetic Tremor—Voluntary Movement Peak Angular Velocity (deg/s) was quantified. In the Wrist Rotation module, the pre-processed angular velocity was integrated (MATLAB’s trapz function) to derive angular position. The Wrist Rotation—Range of Motion Ellipse Area (deg^2^) was calculated with the angular position data using a principal component analysis to define the two major axes of rotation. The mean angular position from each of the two axes defined the major and minor ellipse axes lengths and was used to estimate wrist ROM with the ellipse area formula (pi × a × b). In the Functional Mobility module, bradykinesia during postural transitions, turning, and gait were quantified using angular velocity data from the IMU. The RMS of the resultant angular velocity signal was utilized to characterize the average amplitude of rotational speed throughout the trial [Functional Mobility—Angular Velocity RMS (deg/s)].

#### 2.7.2. Rigidity Metrics

Rigidity symptoms were characterized using kinematic proxies derived from voluntary repetitive movements, based on the premise that rigidity-related motor impairments disrupt the initiation, termination, and smooth sequencing of successive motor programs [31]. Empirical support for this method has been demonstrated in finger tapping metrics during a voluntary repetitive task, which was correlated with clinical measures of rigidity [32]. In the Finger Tapping module, rigidity-related impairments were quantified using the time intervals between successive, alternating finger taps recorded on the tablet [Finger Tapping—Tap Interval (s)], with larger values reflecting slower transitions between the motor programs for the two fingers. In the Kinetic Tremor module, kinematic data near the targets (chin and tablet) were analyzed from the stylus to evaluate the ability of the participant to perform the repetitive task with smooth and rapid transitions between movement termination and initiation. Specifically, all kinematic data were pre-processed using filtering methods described in Section 2.7.1 to isolate voluntary movement from tremor movement. The dwell time or time spent on targets (i.e., tablet or chin) was calculated using the resultant angular velocity signal and used as a proxy for rigidity-related impairments. Specifically, Kinetic Tremor—Voluntary Movement Dwell time (s) and Dwell Time variability [Kinetic Tremor—Voluntary Movement Dwell Time standard deviation (SD) (s)] were used to capture the extent to which movements were performed as fluid, continuous sequences versus discrete movements with prolonged pauses between movement initiation and termination. In addition, the RMS of the resultant acceleration signal [Kinetic Tremor—Voluntary Movement—Linear Acceleration RMS (m/s^2^)] during the repetitive chin-to-tablet task was calculated. Lower RMS values were interpreted as reduced acceleration amplitude at transition points (movement initiations and termination), consistent with diminished fluidity of movement associated with rigidity-related motor impairments.

#### 2.7.3. Tremor Metrics

Tremor symptoms were calculated using the IMU data from the Postural, Resting, and Emergent Tremor modules and the Spiral Drawing module. Briefly, linear acceleration and angular velocity data from each axis of the IMU were filtered using a fourth-order Butterworth band-pass filter (cut-off frequencies of 3.5–12 Hz) to isolate movements in the PD tremor frequency range [29]. For each axis, the power spectral density (PSD) was calculated using Welch’s estimate implemented in MATLAB (window size of 1 s, 50% overlap). The PSDs were combined through summation, and the maximum value of the combined PSD between 3.5 and 12 Hz was determined and used to quantify tremor amplitude. Specifically, postural tremor amplitude was calculated as the maximum value of the linear Acceleration PSD [Postural Tremor—Acceleration Power Peak (m/s^2^)/Hz]. Resting tremor amplitude was calculated as the maximum value of the angular velocity PSD [Resting Tremor—Angular Velocity Peak Power (deg/s)/Hz]. Kinetic tremor amplitude was calculated as the maximum value of the angular velocity PSD during the Spiral Drawing module [Spiral Drawing—Angular Velocity Peak Power [deg/s)/Hz].

Force data recorded from the stylus force transducer during the Emergent Tremor module were utilized to quantify tremor with an Emergent Tremor—Roughness Index (N/s), which was defined as the sum of the absolute value of the derivative of the force signal. A Roughness Index value of zero across the trial duration reflects constant force production (i.e., absence of tremor), whereas higher values indicate increased high-frequency fluctuations in force production consistent with tremor-related movements.

#### 2.7.4. Postural Instability and Gait Difficulty Metrics

Measures of PIGD were calculated from kinematic data recorded with the stylus during the Functional Mobility Assessment. The RMS of the resultant angular velocity signal [Functional Mobility—Axial Angular Velocity RMS (m/s)] and the RMS of the resultant linear acceleration signal [Functional Mobility—Axial Linear Acceleration RMS (m/s^2^)] were used to quantify gait performance. Postural stability during the Standing Balance module was quantified using the 95% sway area [Standing Balance—95% Sway Area (m^2^/s^4^)]. Specifically, a principal component analysis was applied to the linear acceleration signals in the medial–lateral and anterior–posterior plane to calculate the 95% confidence ellipse area [33].

### 2.8. Calculation of Clinical Outcomes

The MDS-UPDRS-III sub-scores were calculated using the items identified in Table 3.

### 2.9. Statistical Analysis

The Consensus-based Standards for the Selection of Health Measurement Instruments (COSMIN) guidelines were followed to establish criterion and known-groups validity of the Ceraxis Insight modules [34,35]. Criterion validity was evaluated through separate mixed effects models and correlation analyses. Mixed-effects models were utilized to determine the relationship between quantitative metrics from the Ceraxis Insight modules and MDS-UPDRS III total scores, and sub-scores (bradykinesia, rigidity, tremor, and PIGD). Dependent variables included the MDS-UPDRS III scores, with subject as a random effect and fixed effects of corresponding quantitative measures (i.e., angular velocity RMS for MDS-UPDRS III bradykinesia sub-score) from the test modules, and DBS group (ON-DBS, OFF-DBS, NO-DBS). The estimate, the standard error of the estimate, and the associated *p*-value from the mixed effects models were reported with *p* < 0.05 as the threshold for statistical significance. In addition, multivariable mixed effects models were constructed to determine the relationship between a combination of quantitative measures from the Ceraxis Insight modules and the total MDS-UPDRS III score. The estimate, the standard error of the estimate, and the associated *p*-value from the mixed effects models were reported with *p* < 0.05 as the threshold for statistical significance. Concordance correlation coefficients (CCC) were reported as a measure of the strength of the linear association between the clinical scores and the predicted clinical scores based on the Ceraxis Insight data, which account for random subject effect, and a score of one describes the perfect relationship [36]. CCC does not perform well when applied to zero-clustered data. For sub-scores with a large number of zeros, such as tremor, Spearman correlation coefficients [37] were also reported as a measure of the strength of the linear association between the clinical scores and the predicted clinical scores based on the Ceraxis Insight data, as they are more robust to zero-inflated data. Due to limitations with repeated measures, Spearman correlation coefficients were reported for the subset of trials in the OFF-DBS or NO-DBS condition. Correlation coefficient values were classified as: moderate (0.40–0.59), strong (0.60–0.79), and very strong (greater than 0.80). Criterion validity was demonstrated when mixed effects models indicated significance at *p* < 0.05, and correlations were strong (CCC or *r* > 0.60) [38].

Secondary analyses evaluated known-groups validity, a type of construct validity, to evaluate the ability of Ceraxis Insight modules to differentiate between clinically relevant groups. Logistic regression models were constructed for participants’ therapy state (ON- versus OFF-DBS therapy). The c-statistic or Area Under the Receiver Operating Characteristic Curve (AUC) was calculated to determine which Ceraxis Insight metrics could discriminate PwPD based on therapy state (ON- and OFF-DBS therapy) for the subset of PwPD with DBS (*n* = 8). AUC values were classified as: acceptable (0.70–0.79), excellent (0.80–0.89), and outstanding (greater than 0.90). Known-groups validity was established based on pre-specified hypotheses that AUC values would be at least acceptable (i.e., >0.70).

## 3. Results

Nineteen PwPD completed the COMMAND study off-antiparkinsonian medication. A subset of eight PwPD with DBS was tested in both the ON-DBS and OFF-DBS conditions. In total, 27 datasets were analyzed (Table 1).

### 3.1. Cohort Included a Wide Range of PD Symptoms and Disease Severities

The mean MDS-UPDRS-III score for all participants (*n* = 19) off therapy was 35.2 (17.8). Participants with DBS demonstrated a 38% improvement in the ON-DBS condition compared to the OFF-DBS condition. Across all 27 assessments, the span of motor symptoms and dysfunction provided a broad range [min, max] of severities: MDS-UPDRS III total score [13, 84], and bradykinesia [2, 39], rigidity [0, 14], tremor [0, 17], and PIGD [0, 14] sub-scores.

### 3.2. Criterion Validity: Ceraxis Insight Measures Strongly Correlated with MDS-UPDRS III Total and Sub-Scores

Overall, the Ceraxis Insight modules demonstrated criterion validity with the MDS-UPDRS III sub-scores. A subset of the results is presented in Table 4 with additional metrics provided in Supplementary Materials.

#### 3.2.1. Bradykinesia

Two measures from the Ceraxis Insight modules predicted clinical measures of bradykinesia [13]. Specifically, decreases in the angular velocity during the Functional Mobility module (Angular Velocity RMS; −0.39, (0.08), *p* < 0.01) and decreases in linear acceleration of voluntary upper extremity movements during the Kinetic Tremor module (Voluntary Movement—Linear Acceleration RMS; −1.90, (0.77), *p* < 0.05) significantly predicted increases in bradykinesia sub-score. The correlation between Bradykinesia clinical scores and predicted scores based on the Ceraxis Insight data was strong for both metrics (*r* = 0.87).

#### 3.2.2. Rigidity

Two Ceraxis Insight modules produced kinematic measures that significantly predicted clinical measures of rigidity. Specifically, increases in the tap interval during voluntary movements in the Finger Tapping module (Voluntary Movement—Tap Interval; 11.81 (4.7), *p* < 0.05) and decreases in the RMS of linear acceleration during voluntary movements in the Kinetic Tremor module (Voluntary Movement—Linear Acceleration RMS; −1.10 (0.39), *p* < 0.05) corresponded with increasing measures of rigidity. These findings were supported by a strong correlation between clinical scores and predicted scores of rigidity (*r* = 0.81 and 0.82, respectively).

#### 3.2.3. Tremor

Two kinematic measures from the Ceraxis modules, Postural Tremor (Acceleration Peak Power; 0.85 (0.23), *p* < 0.01), and Emergent Tremor (Tremor Roughness Index; 0.001 (0.0002), *p* < 0.023) significantly predicted clinical measures of tremor. Predicted measures of postural and emergent tremor based on the Ceraxis modules demonstrated strong (*r* = 0.68) and very strong positive (*r* = 0.89) correlation with clinical measures, respectively.

#### 3.2.4. Postural Instability and Gait Dysfunction

Clinical measures of PIGD were significantly related to linear acceleration (Axial Linear Acceleration RMS; −2.72 (1.08), *p* < 0.05) and angular velocity (Axial Angular Velocity RMS; −0.13 (0.03), *p* < 0.05) during the Functional Mobility module. Specifically, Ceraxis Insight acceleration and speed decreased as PIGD sub-scores worsened, and these results were supported by the concordance correlation coefficients value (*r* = 0.77) and (*r* = 0.80), respectively.

#### 3.2.5. Overall Disease Severity

A multivariable mixed-effects model was utilized to determine the relationship between quantitative measures derived from the Ceraxis Insight modules and the total MDS-UPDRS III score, a measure of overall motor symptom severity. The MDS-UPDRS III total score was best predicted with a measure of bradykinesia (Wrist Rotation—Range of Motion Ellipse Area; −0.001 (0.0002), *p* < 0.008) and (Kinetic Tremor—Voluntary Movement—Peak Angular Velocity; −0.07 (0.02), *p* < 0.028) and postural stability (Functional Mobility—Axial Angular Velocity RMS; −0.63 (0.13), *p* < 0.008) (Table 4).

### 3.3. Known-Groups Validity: Ceraxis Insight Metrics Discriminate PwPD Based on DBS Condition

**Figure 2:**
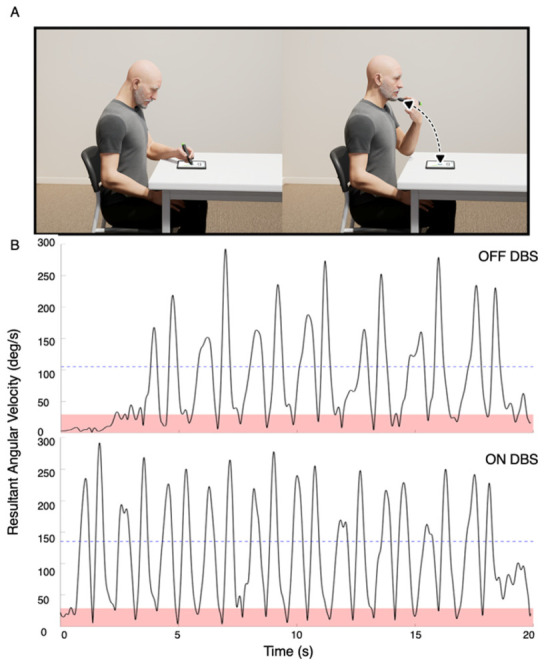
(**A**) Illustration of a participant performing the Kinetic Tremor module. The assessment requires the participant to perform a repetitive task, touching the tip of the Ceraxis Insight Motiv Stylus to their chin and then to an icon on the tablet screen while kinematic data is recorded from the stylus IMU. (**B**) Representative resultant angular velocity data (black solid line) in the OFF-DBS (upper plot) and ON-DBS (lower plot) condition for one participant. The blue dashed line indicates the average amplitude of angular velocity (deg/s) for each trial (i.e., Angular Velocity RMS). The local minima represent target touches (chin or tablet). The red shaded region is 0–10% of the maximum angular velocity and represents slow movements near target touches. The time during which the angular velocity trace was within the shaded pink area represents Dwell Time.

Representative resultant angular velocity data during the Kinetic Tremor module from one participant tested OFF- and ON-DBS demonstrates significant differences between therapy states (Figure 2). Visual inspection reveals impairments in motor performance, evidenced by fewer Number of Chin/Tablet Touches (fewer local minimums) and reduced angular velocity magnitude (lower Angular Velocity RMS (blue dashed line)) and reduced rhythmicity (i.e., increased variability in time spent on targets (Dwell Time SD) in the OFF-DBS compared to ON-DBS condition).

Similar differences were demonstrated between the ON- and OFF-DBS conditions across several Ceraxis Insight metrics. Specifically, the AUC was calculated to quantify the ability of these metrics to classify participants (*n* = 8) by DBS-therapy condition. Eleven Ceraxis Insight metrics successfully discriminated between ON- and OFF-DBS conditions as demonstrated by acceptable to excellent AUC values (AUC > 0.7, Table 5). Notably, three modules, Resting Tremor, Spiral Drawing, and Standing Balance, did not correlate significantly with MDS-UPDRS III scores but demonstrated AUCs ranging from 0.72 to 0.81, indicating acceptable to excellent discrimination of PD motor symptoms beyond those captured in the MDS UDPRS-III (Table 5).

### 3.4. System Usability Scale Results

The System Usability Scale is a questionnaire in which subjects self-report the usability of a system by rating 10 statements on a five-point Likert scale, from strongly disagree (0) to strongly agree (4). The System Usability Scale has a maximum score of 100, with 68 being the average, and a score of 78.8 or greater earns an “A” grade (top 15 percentile of systems) [39]. The average System Usability Score for the Ceraxis Insight platform across all participants was 84.8.

## 4. Discussion

The Ceraxis Insight platform successfully demonstrated criterion validity, providing metrics that significantly predicted and correlated with the current gold-standard clinical assessment, the MDS-UPDRS III, across the cardinal motor symptoms of PD—tremor, bradykinesia, rigidity, and PIGD and overall disease severity. Specifically, the six modules from the platform captured kinematic and kinetic metrics that significantly predicted the clinical ratings of a movement disorders neurologist. Five of these modules also demonstrated the ability to discriminate between ON- and OFF-DBS therapy conditions. In addition, three other modules demonstrated the ability to discriminate between ON- and OFF-DBS therapy conditions but did not correlate with clinical measures, suggesting these modules captured additional information beyond the MDS-UPDRS III. The Ceraxis Insight metrics were obtained through a self-administered assessment, with high usability ratings, that took less than 30 min to complete and provided high-resolution data on PD motor symptom severity without the clinical oversight required from the current gold-standard evaluation. Collectively, these results support the use of the Ceraxis Insight platform as a comprehensive, valid tool for quantifying and tracking PD symptoms that can be leveraged for detailed, objective assessment to better inform clinical decision making.

Traditional clinical assessments, like the MDS-UPDRS III, evaluate symptoms in isolation, despite their interconnectedness. For example, cogwheeling, a type of rigidity that is elicited during both passive and voluntary movements, has been associated with tremor-related impairments, as evidenced by significant correlations between clinical and quantitative measures of cogwheel rigidity and tremor [40,41]. Previous data also highlight a connection between rigidity and bradykinesia, showing that (1) the long-latency stretch reflexes related to rigidity may contribute to bradykinesia in the antagonist muscle during agonist activation, (2) there is a strong correlation between the clinical measures of rigidity and bradykinesia, (3) both symptoms demonstrate a similar response to traditional therapies used to treat PD, and (4) both symptoms show a similar correlation to the pathological neural signals that are associated with PD [42,43,44,45]. Results from this study support these findings as the same kinematic outcome during voluntary movement captured with the Ceraxis Insight platform (i.e., Kinetic Tremor—Voluntary Movement Linear Acceleration RMS) was significantly correlated to both clinical measures of bradykinesia and rigidity, suggesting that the underlying mechanisms of rigidity that impair passive movements may also impact components of voluntary movement. In addition, Trager and colleagues utilized an alternating finger tapping paradigm, similar to the Ceraxis Insight Finger Tapping module, to identify kinematic metrics that were correlated to MDS-UPDRS III rigidity [32]. Notably, Trager and colleagues utilized a highly calibrated musical keyboard in their paradigm. In this study, Tap Interval, or the time between alternating finger taps captured on the touchscreen of a commercially available iPad, showed a significant relationship to clinical measures of rigidity. Using the technology in the Ceraxis Insight platform to capture objective, high-resolution measurements of movements may provide greater insight into the interconnectedness between the cardinal motor symptoms of PD and offer the additional benefit of capturing impairments across multiple domains in one module. For example, MDS-UPDRS III item 3.16 (kinetic tremor of the hands) is scored solely on the highest tremor amplitude observed during a finger-to-nose maneuver. By contrast, the Ceraxis Insight Kinetic Tremor module revealed symptoms of rigidity and bradykinesia. Furthermore, capturing an objective, comprehensive picture of the PD symptoms, including measures correlated to rigidity, through a self-administered assessment addresses the limitations of existing technologies that evaluate a single symptom or are unable to quantify rigidity symptoms, and facilitates more frequent evaluation of motor symptoms to better inform treatment decisions.

Symptoms of PD fluctuate hour-to-hour and day-to-day, affected by stress and fatigue, as well as the short half-life of many PD medications [46,47,48,49]. Therefore, even for PwPD seen and evaluated by an MDN routinely, optimal care is impeded, as the range and variability of symptoms are not captured in this brief snapshot. To mitigate this limitation, various remote devices have been developed to elucidate patients’ symptomology beyond just the doctor’s office [50,51,52,53]. These efforts have demonstrated the feasibility of at-home assessment, but the devices and their data are inherently limited. First, none have been shown to validly capture all four cardinal motor symptoms of PD, so while they may provide a snapshot of tremor or bradykinesia symptoms, they do not capture the nuances of symptoms and their interconnectedness [54]. Additionally, many of these devices need to be worn for an extended period of time, a physical burden that PwPD may be reluctant to embrace or provide hour-by-hour binary data on the presence or absence of a symptom [55]. However, the Ceraxis Insight assessment can be completed in less than half an hour and does not require participants to wear a device all day. The correlations between Ceraxis Insight metrics and each of the four PD cardinal motor symptoms show that this assessment captures valid measures of bradykinesia, tremor, rigidity, and PIGD and accurately discriminates between therapy states. Notably, this is one of the first demonstrations of validated rigidity measures from a self-administered task, without the hands-on passive manipulation of a clinician [32]. Considering the validity of multiple metrics for each PD motor symptom and the portability and ease of use, the Ceraxis Insight platform provides a model to provide clinicians with comprehensive, objective data with minimal impediments to the daily lives of PwPD.

Despite the promising results of this study, further investigation is warranted. While a sample of 19 PwPD was adequate to demonstrate significant correlations to the MDS-UPDRS III sub-scores and sensitivity to DBS condition in this sample, a larger cohort with a control group and a broader range of PD-symptom severities is needed to advance clinical adoption of the Ceraxis Insight platform. Similarly, the present study had inclusion criteria of being able to independently walk 200 m, which was implemented to ensure participants could complete all assessments, but limited the cohort to ~10% of participants with “severe” PD impairment. Future studies will evaluate the generalizability of the exploratory results of this study to more severely impaired individuals, as well as those with various PD subtypes, such as tremor- or PIGD-dominant. All assessments were completed in the off-medication state. Future studies also need to assess the validity of the platform across several levels of impairment, from mild and early stage to the most advanced. All testing for this study was conducted in a clinical setting. Further investigation is planned to assess the feasibility of remote assessment.

Notably, the MDS-UPDRS III was selected as the criterion for this study because of its central role in the clinical evaluation of PD symptoms, despite recognized limitations in sensitivity and inter-rater reliability [14,15]. The scale’s ordinal, clinician-rated 0–4 scoring system limits its ability to detect subtle changes in motor performance, introduces a degree of subjectivity into symptom assessment, and produces an established floor effect [56]; the maximum rating of four for each item may also introduce a theoretical ceiling effect (e.g., rest tremors of 11 and 20 cm in amplitude, respectively, each is scored as a 4). While the floor and ceiling effects of the Ceraxis Insight metrics were not analyzed in this study, the continuous, unbounded data may mitigate the limitations of the MDS-UPDRS III and will be analyzed with a larger cohort in future studies.

Ultimately, even with the development of technologies to measure PD symptoms, access to specialized care for PwPD is inadequate. Despite clinical recommendations for regular visits to an MDN for the clinical management of PD, recent data show that only 9% of PwPD have an annual visit with a specialist, and over 40% are not seen by any neurologist [10,16,57]. Therefore, a user-friendly platform that provides detailed, clinically relevant data on PD symptoms is critical for expanding the availability of validated, comprehensive assessments of PD motor symptoms, and ultimately, access to optimal clinical care to the disappointingly large population of underserved PwPD. To address the discouragingly low utilization of and access to high-quality, PD-specific care, a web portal has been integrated into the Ceraxis Insight platform. This allows clinicians to “prescribe” assessments, which PwPD can perform independently at home, and have the data uploaded for review by an MDN.

Testing the validity of Ceraxis Insight metrics against MDN clinical ratings and the ability to discriminate between therapy states was a critical step in our ‘develop with clinical intent’ approach [23]. Thus, this study represents a critical first step in advancing the Ceraxis Insight system for integration into the clinical workflow and for patient-administered, comprehensive remote assessment of PD motor symptoms, including fine-motor, axial, and postural and gait impairments. The Ceraxis Insight platform may enable clinicians to leverage detailed, objective data to provide quality care and manage PD symptoms, including monitoring and adjusting response to DBS and time-sensitive medications, and address the substantial unmet need for specialized PD care.

## 5. Conclusions

The Ceraxis Insight platform captured objective, quantitative data on the cardinal motor symptoms of PD. Unlike previous technologies, the self-administered, user-friendly Ceraxis Insight platform provides a comprehensive picture of PD motor symptoms and may advance progress toward automating and digitizing the MDS-UPDRS III. Considering that a significant population of PwPD are underserved and do not have access to clinical-standard PD-focused medical care, this technology has the potential to significantly improve the clinical care and quality of life for those who may otherwise remain disadvantaged.

## Figures and Tables

**Table 1:** Participant demographics.

Age at Enrollment	
Mean (SD)	66.6 (6.97)
Sex	
Female	7 (36.8%)
Male	12 (63.2%)
Education Level	
College Degree	8 (42.1%)
High School	6 (31.6%)
Master’s Degree	3 (15.8%)
PhD	1 (5.3%)
Not Disclosed	1 (5.3%)
Number of Falls in Past 6 Months	
Mean (SD)	0.684 (1.63)
Employment Status	
Employed	9 (47.4%)
Not Employed	2 (10.5%)
Retired	8 (42.1%)
MDS-UPDRS-III Total (Off Therapy)	
Median [Min, Max]	33.0 [17.0, 84.0]
Bradykinesia Sub-Score	
Median [Min, Max]	12.0 [3.00, 39.0]
Tremor Sub-Score	
Median [Min, Max]	7.00 [0, 17.0]
Rigidity Sub-Score	
Median [Min, Max]	6.00 [1.00, 14.0]
Postural Instability and Gait Difficulty Sub-Score	
Median [Min, Max]	3.00 [0, 11.0]
Disease Severity * (Off Therapy)	
Mild (count)	9
Moderate (count)	8
Severe (count)	2

* Disease severity was classified using the scale developed by Martinez-Martin et al. [26].

**Table 2:** Ceraxis Insight modules and their corresponding reference clinical measures.

Ceraxis Insight Module	Module Description	Reference Clinical Measures
1. Kinetic Tremor	The participant holds the stylus like a pen and repeatedly taps the tip between the chin and a target circle on the tablet screen	MDS-UPDRS III 3.16: Kinetic Tremor of the Hands
2. Postural Tremor	The participant holds the stylus in a closed fist, with palms down, and arms stretched out in front of the body	MDS-UPDRS III 3.15: Postural Tremor of the Hands
3. Resting Tremor	The participant holds the stylus in a closed fist, with both hands resting on the lap	MDS-UPDRS III 3.17: Rest Tremor Amplitude
4. Wrist Rotation	The participant holds the stylus in a closed fist and rotates the wrist in a circular motion	MDS-UPDRS III 3.6: Pronation-Supination Movements of the Hands
5. Spiral Drawing	The participant holds the stylus like a pen and traces a spiral shown on the screen	Archimedes Spiral Task
6. Emergent Tremor	The participant holds the stylus like a pen and presses the tip onto the tablet screen, maintaining a target amount of pressure	Dot Approximation Task
7. Finger Tapping	The participant performs a repetitive, alternating tapping task with their index and middle fingers on two icons displayed on the tablet screen	MDS-UPDRS III 3.4: Finger Tapping
8. Standing Balance	The participant stands with their feet together, and their eyes open and then closed, with the stylus positioned in a waist-worn holster	MDS-UPDRS III 3.12: Postural Stability
9. Functional Mobility	The participant stands up from a chair, walks 3 m, turns 180 degrees, walks back to the chair, turns 180 degrees, and sits in the chair with the stylus positioned in a waist-worn holster	MDS-UPDRS III 3.9: Arising from Chair MDS-UPDRS III 3.10: Gait Timed Up and Go

**Table 3:** MDS-UPDRS III items and their associated clinical variables.

Clinical Sub-Score	MDS-UPDRS III Components
Bradykinesia Total	3.2, 3.4–3.8
Rigidity Total	3.3
Tremor Total	3.15–3.18
PIGD Total	3.9–3.13

**Table 4:** Criterion validity: mixed effects models of Ceraxis Insight metrics predicting MDS-UPDRS III sub-scores and total score.

MDS-UPDRS III Sub-Score	Ceraxis Insight Module	Metric	Estimate (SE)	*p*-Value	Concordance Correlation	Spearman Correlation
					*n* = 27	*n* = 19 (NO-DBS, OFF-DBS)
Bradykinesia	Functional Mobility	Axial Angular Velocity RMS	−0.39 (0.08)	0.003	**0.87**	0.47
	Kinetic Tremor	Voluntary Movement—Linear Acceleration RMS	−1.90 (0.77)	0.048	**0.87**	0.57
Rigidity	Finger Tapping	Voluntary Movement—Tap Interval	11.81 (4.70)	0.046	**0.81**	0.48
	Kinetic Tremor	Voluntary Movement—Linear Acceleration RMS	−1.10 (0.39)	0.029	**0.82**	0.46
Tremor	Postural Tremor	Tremor—Linear Acceleration Peak Power	0.85 (0.23)	0.01	0.23	**0.68**
	Emergent Tremor	Roughness Index	0.001 (0.0002)	0.023	0.23	**0.89**
PIGD	Functional Mobility	Axial Linear Acceleration RMS	−2.72 (1.08)	0.045	**0.77**	0.54
	Functional Mobility	Axial Angular Velocity RMS	−0.13 (0.03)	0.008	**0.80**	0.71
MDS-UPDRS III Total	Kinetic Tremor	Voluntary Movement—Peak Angular Velocity	−0.07 (0.02)	0.028	**0.62**	**0.87**
	Wrist Rotation	Range of Motion—Ellipse Area	−0.001 (0.0002)	0.008		
	Functional Mobility	Axial Angular Velocity RMS	−0.63 (0.13)	0.008		

Bolding indicates strong to very strong correlations. Metrics labeled as voluntary were derived from kinematic data that were band-pass filtered (0.25–3.5 Hz) to isolate voluntary movements, and metrics that include tremor were band-pass filtered (3.5–12 Hz) to isolate tremor.

**Table 5:** Known-groups validity (*n* = 8). Ceraxis Insight modules are sensitive to DBS-therapy conditions.

Ceraxis Insight Module	Metric	AUC *
		(ON-DBS vs. OFF-DBS)
Kinetic Tremor	Voluntary Movement—Number of Chin/Tablet Touches ^+^	**0.91**
Kinetic Tremor	Voluntary Movement—Angular Velocity RMS ^+^	**0.83**
Functional Mobility	Axial Angular Velocity RMS	**0.82**
Kinetic Tremor	Voluntary Movement—Dwell Time SD ^+^	**0.81**
Resting Tremor	Tremor—Angular Velocity Peak Power ^^^	**0.81**
Emergent Tremor	Roughness Index	**0.75**
Finger Tapping	Voluntary Movement—Tap Interval ^+^	**0.73**
Postural Tremor	Tremor—Linear Acceleration Peak Power ^^^	**0.72**
Kinetic Tremor	Voluntary Movement—Linear Acceleration RMS ^+^	**0.72**
Spiral Drawing	Tremor—Angular Velocity Peak Power ^^^	**0.72**
Standing Balance	95% Sway Area	**0.72**

* Classification of AUC values: acceptable (0.70–0.79); excellent (0.80–0.89); outstanding (greater than 0.90). ^+^ Metrics labeled as voluntary were derived from kinematic data that have been band-pass filtered (0.25–3.5 Hz) to isolate voluntary movements^. ^^ Metrics that include tremor have been band-pass filtered (3.5–12 Hz) to isolate tremor movement.

## Data Availability

The data presented in this study are available on request from the corresponding author due to legal and privacy restrictions.

## Supplementary Materials

The following supporting information can be downloaded at https://www.mdpi.com/article/10.3390/s26144497/s1: Table S1: Criterion validity: additional Ceraxis Insight—MDS-UPDRS III correlations.
