# Supplementary material for: A Self-Administered, Digitized Approach to Quantifying the Cardinal Motor Symptoms in Parkinson’s Disease
Source: Sensors (Basel). 2026 Jul 15;26(14):4497. doi: 10.3390/s26144497 (PMC13419166; doi:10.3390/s26144497)
Supplement: Supplementary file 1 [file sensors-26-04497-s001.zip › sensors-4348844-supplementary.pdf]

Supplementary Materials:

Supplementary Table S1. Criterion Validity: Additional Ceraxis Insight—MDS-UPDRS III Correlations.

MDS-UPDRS III Sub-score	Ceraxis Insight Module	Metric	Estimate (SE)	*p*-value	Concordance Correlation	Spearman Correlation
					n = 27	*n* = 19 (NO-DBS, OFF-DBS)
**Bradykinesia**	Functional Mobility	Sit-To-Turn Mean Velocity (deg/s)	-0.16 (0.04)	0.007	**0.87**	0.48
	Wrist Rotation	Voluntary-ROM Ellipse Area (deg^2)	-0.0005 (0.0001)	0.006	**0.88**	0.63
**Rigidity**	Wrist Rotation	Voluntary-ROM Ellipse Area (deg^2)	-0.0002 (0.0001)	0.010	**0.83**	0.52
	Functional Mobility	Angular Velocity RMS (deg/s)	-0.17 (0.05)	0.016	**0.81**	0.27
	Kinetic Tremor	Voluntary- Peak Angular Velocity (deg/s)	-0.05 (0.02)	0.031	**0.75**	0.50
	Functional Mobility	Sit-To-Turn Peak Velocity (deg/s)	-0.06 (0.02)	0.029	**0.81**	0.41
**Tremor**	Postural Tremor	Angular Velocity RMS (deg/s)	0.06 (0.02)	0.037	0.18	**0.70**
	Postural Tremor	PTA-Acceleration RMS (m/s^2)	2.50 (0.64)	0.008	0.26	**0.70**
**PIGD**	Functional Mobility	Sit-to-turn Mean Velocity (deg/s)	-0.04 (0.02)	0.041	**0.79**	0.62
